# Sample size in social contact surveys for epidemic modelling and risk prediction

**DOI:** 10.1017/S095026882610212X

**Published:** 2026-10-15

**Authors:** Ellen Brooks-Pollock, Leon Danon

**Affiliations:** 1 Population Health Sciences, Bristol Medical School, University of Bristol, UK; 2 School of Engineering Mathematics and Technology, University of Bristol, UK; 3 Jean Golding Institute, University of Bristol, UK

**Keywords:** contact matrix, epidemic model, infectious disease, POLYMOD, reproduction number, sample size, social contact survey, survey methodology

## Abstract

Social contact surveys, measuring who contacts whom, are widely used for epidemic intelligence and public health decision-making. Despite their widespread use, sample size calculations are not performed. We conducted a review of social contact surveys (2008–2025) to characterize current practice. We characterized the impact of survey size on epidemic metrics using two social contact surveys, the UK Social Contact Survey and POLYMOD (Europe), and two methods. For each dataset and approach, we generated repeated subsamples and calculated the resulting reproduction numbers, characterized their distributions, and measured uncertainty. We identified 107 unique social contact surveys from 57 studies. Sample sizes ranged from 30 to more than 10 000 participants, with a median of 1 438. One quarter of surveys contained fewer than 1 000 participants. Our analysis supports recruiting at least 1 000 participants for general-population social contact surveys used for epidemic modelling. Increasing sample size to 2 000 participants provides worthwhile gains in the precision of epidemic estimates, particularly for analyses using finer age stratification or individual-level approaches. Beyond approximately 3 000 participants, additional improvements in precision were small for the epidemic metrics analysed. This provides a practical balance between precision and participant burden.

## Key Results

- There is no consensus on the number of participants needed in a social contact survey used for epidemic modelling.
- We found that the number of participants sampled impacts epidemic predictions: recruiting at least 1 000 and up to 3 000 participants balances precision and participant burden.

This work provides a practical, evidence-based target for future social contact surveys used for epidemic intelligence and public health decision-making.

## Introduction

Social contact surveys have become a crucial data source for estimating real-time epidemic risk in a population and informing structured infectious disease models [1, 2]. Prior to the POLYMOD study of age-stratified social contact patterns across eight European countries [3], it was often assumed that obtaining accurate empirical data on ‘*who mixes with whom’* at scale would be prohibitively difficult, given ambiguities in defining what constitutes a ‘contact’ and the burden of detailed recording [4]. POLYMOD demonstrated both the feasibility of collecting contact diary data and the presence of robust, highly structured age-stratified mixing patterns that were remarkably consistent across countries, validating the value of such data for transmission modelling and vaccination policy design. Following this, social contact surveys have been implemented in multiple settings and time periods, including during the 2009 influenza and COVID-19 pandemics [5, 6], where they were used to measure behavioural change and the impact of non-pharmaceutical interventions on transmission.

Social contact data can be obtained using questionnaire-based surveys (paper, online, or interviewer-administered), measured through proximity sensors or mobile phones, or inferred indirectly from demographic, census or household composition data, each with trade-offs between logistical feasibility, recall bias and the level of detail on contact type and setting [1, 7, 8]. The most common format for questionnaire-based social contact surveys is to ask participants to list their social contacts on the previous day, usually distinguishing between physical and conversational contacts and collecting information on age, setting, frequency, and duration. Survey instruments have been refined over time to reduce respondent burden and improve data quality, for example, by allowing participants to report groups of similar contacts (such as an entire school class) rather than enumerating each individual separately [9, 10]. Despite these improvements, completing a social contact diary can remain time-consuming, particularly for individuals with many daily contacts, contributing to non-response, partial completion, and dropout in longitudinal designs. Qualitative work has highlighted the importance of participants’ understanding of the survey’s purpose for eliciting accurate responses and sustaining engagement over time [11–13].

Traditional sample size calculations in epidemiological studies typically rely on specifying an expected effect size, the variability of the outcome, and the desired power and significance level [14]. However, these approaches are not immediately applicable to social contact surveys, which are often observational, do not test a single, pre-specified hypothesis, and aim instead to characterize complex contact patterns that feed into model-based estimates such as the reproduction number. In addition, social contact surveys frequently face practical constraints, including limited participation rates, survey fatigue, and potential bias towards responding on days with fewer contacts [15], all of which complicate the relationship between sample size and the precision of derived epidemiological quantities. As a result, sample sizes in published social contact surveys have typically been determined by pragmatic considerations such as budget, recruitment channels, and logistics, rather than by formal power calculations.

Information and data from social contact surveys underpin epidemic analyses, model projections, and policy decisions [1, 3, 16]. Increasing sample size is costly and burdensome for participants and raises ethical questions if diaries are unnecessarily long or frequent without proper justification. Simple hypothesis-testing approaches to sample size, based on detecting differences in mean contact rates between groups, typically suggest smaller samples and do not reflect how these data are used in transmission models. Here, we seek to determine a practical target sample size for social contact surveys. We first conducted a rapid review of published contact surveys to characterize current practice in participant numbers, then used UK Social Contact Survey (UKSCS) [9, 10] and POLYMOD to quantify how survey size affects uncertainty in estimates of the reproduction number, and used this to provide a pragmatic estimate.

## Aim

The aims of this study were to review current practice for social contact survey sample size and to use epidemic metrics to determine a practical target size for social contact surveys used in epidemic modelling.

## Methods

### Review of current practice

We searched PubMed for all published articles written in English and published between 1 January 2008 and 11 March 2025, using the search string:

$$\begin{array}{l} \left(\left(\text{social contact}*\left[\text{Title}/\text{Abstract}\right]\right)\text{AND}\left(\text{survey}\left[\text{Title}/\text{Abstract}\right]\right)\right)\\ \text{AND}\left(\text{infectio}*\left[\text{Title}/\text{Abstract}\right]\right). \end{array}$$

Studies were included if they met all of the following criteria: (i) primary reports of social contact data (secondary analyses were excluded), (ii) surveys that included questions specifically addressing the number of in-person social contacts, (iii) studies pertaining to human populations (surveys measuring contact patterns exclusively in animals, or human–animal contact patterns, were excluded), (iv) infectious disease focus, and (v) surveys encompassing any age group or demographic. This search string is contained within previous systematic reviews [1, 2], with the addition of the requirement to be related to infections and without any country requirements.

The first author screened titles and abstracts against the inclusion criteria and reviewed full texts where necessary to determine eligibility. Reference lists of included full texts were searched for additional studies missed by the initial search strategy; these were included if they met the inclusion criteria.

From each included study, we extracted the number of participants and the survey setting or country. Descriptive analyses were conducted on the extracted sample sizes.

### Sensitivity of epidemic measures to social contact sample size

To explore the impact of sample size on epidemic measures, we used data from two commonly used social contact surveys and used two methods for estimating the reproduction number from social contact data.

#### POLYMOD

POLYMOD was an eight-country European social contact survey conducted in 2005 [3]. They surveyed 7 290 individuals on their social contacts for the preceding day. Demographic characteristics of the contacts included age, gender, and social context (home, work, school, and other). POLYMOD is the most widely used social contact survey for epidemic modelling [1]. Data are typically incorporated into models by reducing the data to an age-stratified contact matrix which is used within an age-structured compartmental model. For the purposes of this study, we used data from all eight countries to create an age-stratified mixing matrix in 10-year age bands. We calculated the population reproduction number as the dominant eigenvalue of the age-stratified contact matrix, normalized to the sample mean [17, 18].

#### UK Social Contact Survey (UKSCS)

UK Social Contact Survey (UKSCS) was conducted in 2010 and surveyed 5 861 individuals using a paper and online survey [9, 10]. The UKSCS also asked individuals to record their social contacts on the preceding day, but with an additional option to report groups of individuals in order to capture individuals with high numbers of contacts [15]. As age of contact was not recorded in the UKSCS, we employed a previously published method for calculating the population-level reproduction number under the proportionate mixing assumption [16, 19]. Briefly, an adjacency matrix is constructed which is equivalent to a mixing matrix where each entry represents contact between two individuals. Under the assumption that the probability of contacting person j is proportional to j ’s total contacts, $c_{j}$ , divided by all contacts in the population, $\sum_{j}c_{j}$ , the $\left(i,j\right)\mathrm{th}$ entry in the adjacency matrix is $c_{i}c_{j}/\sum c_{j}$ . As this matrix is symmetric, the maximum eigenvalue of the adjacency matrix is proportional to the sum of squared individual contact numbers, since each individual’s probability of both acquiring and transmitting infection scales with their contact rate [16]. Because there is no aggregation across individuals, this approach captures individual-level heterogeneity. The reproduction numbers are normalized to the sample mean.

We repeated the analysis for social contact data from Hong Kong (2015/2016) [20] in the Supplementary Material.

### Simulating social contact survey sample size

We simulated social contact surveys of varying size by drawing repeated subsamples from the full datasets. Sample sizes ranged from 100 to the maximum number of participants; for each, we drew 1 000 random samples with replacement. For each simulated sample size, we calculated the mean, range, and standard deviation in the resulting normalized reproduction numbers, and the Kolmogorov–Smirnov (KS) statistic as the maximum difference between the cumulative distribution of reproduction numbers compared to a sample of 5 800 individuals (close to the full UKSCS sample), together with the associated *p*-values, calculated using ks. test in R. The KS statistic varies between 0 (limited difference between distributions) and 1 (larger difference between distributions). A small associated *p*-value indicates that the two samples follow different distributions. We interpret the statistics in combination to identify a guide for suitable survey sizes.

## Results

The initial search for social contact surveys related to infectious disease transmission returned 145 records. Following title and abstract screening, 93 studies were taken forward for full-text review. The most common reasons for exclusion were: not a contact survey (*n* = 28), not related to infectious disease transmission (*n* = 23), and secondary analyses (*n* = 32). In addition, two studies were identified through reference list searching, resulting in 95 studies being assessed for review. After full-text review, 57 studies were included in the final analysis. Because several papers reported more than one survey or setting, these 57 studies corresponded to 107 unique social contact surveys.

There was a wide range in reported sample sizes across the 107 surveys (Figure 1). The smallest was a pilot study of 30 participants, while the largest exceeded 10 000 – five surveys of this scale had been embedded within larger national studies, such as a census. Smaller surveys tended to focus on specific subgroups, such as university students. One quarter of all surveys contained fewer than 1 000 participants, and three-quarters contained fewer than 2 500 participants. The median sample size was 1 438 participants.

**Figure 1.**
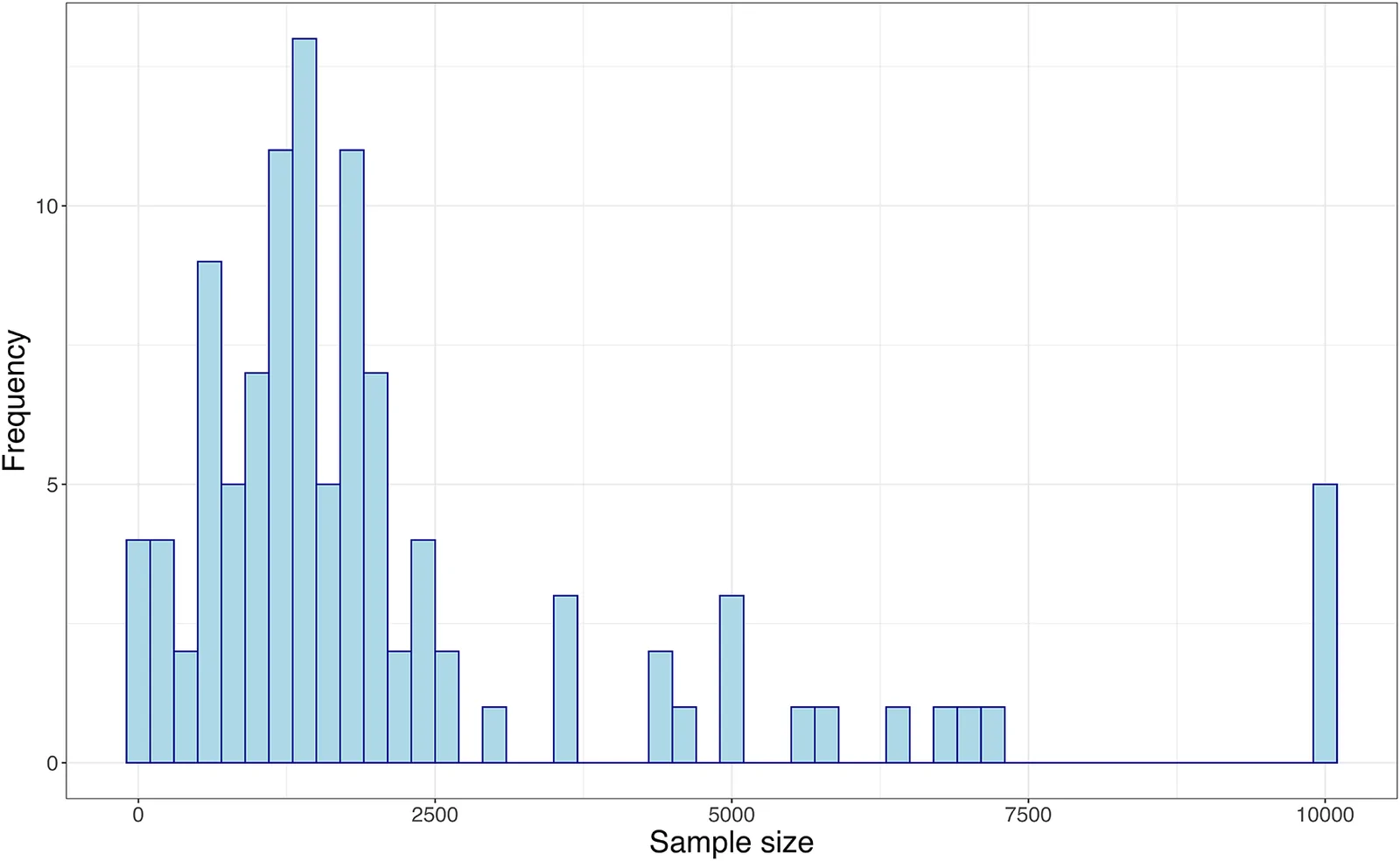
Distribution of social contact survey sizes from a rapid review of 107 surveys. Figure 1. long description.

### Impact of sample size on estimates of the reproduction number

To assess how social contact survey sample size influences epidemic metrics, we analysed two widely used datasets and applied two methods to estimate the population-level reproduction number. Across both datasets and all methods, uncertainty in the reproduction number decreased with sample size (Figure 2; Table 1). Estimates derived using the dominant eigenvalue approach with POLYMOD data and 10-year age bands showed consistently lower uncertainty than those obtained using the sum of squared individual transmission potential with UKSCS data.

**Figure 2.**
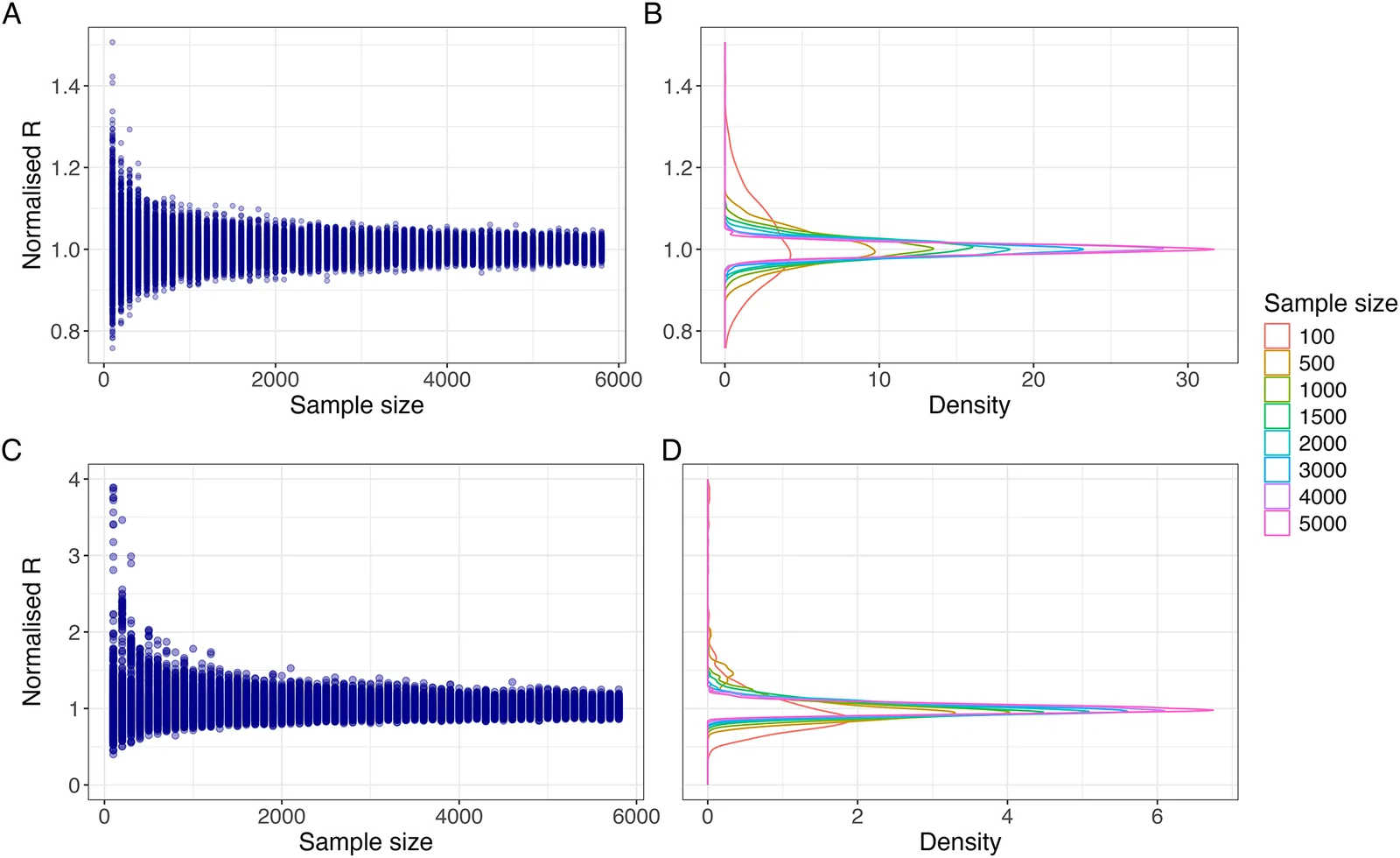
Relationship between number of participants contributing social contact data (sample size) and estimated reproduction number. Panels (a) and (b) show the normalized dominant eigenvalue of the next-generation matrix derived from the POLYMOD study; panels (c) and (d) represent the normalized reproduction number derived from the UKSCS. The points in panels (a) and (c) show individual values derived from a single sample, while panels (b) and (d) summarize the distributions at specific values of the sample size shown as different colours. Figure 2. long description.

**Table 1.** Summary statistics of the estimated reproduction number, normalized to the value at a sample size of 5 800, as a function of social contact survey sample size Table 1. long description.

Sample size	UK Social Contact Survey normalized R				POLYMOD normalized R			
	Mean (min, max)	S.D.	K–S D.	*p*-value	Mean (min, max)	S.D.	K–S D.	*p*-value
200	1.00 (0.50, 3.46)	0.311	0.42	4.91e–77	1.01 (0.82, 1.26)	0.066	0.33	7.13e–49
500	1.01 (0.65, 2.03)	0.198	0.31	2.34e–41	1.00 (0.89, 1.12)	0.042	0.28	1.87e–35
1 000	1.00 (0.72, 1.78)	0.130	0.24	3.12e–25	1.00 (0.91, 1.11)	0.031	0.24	2.79e–26
1 200	1.00 (0.76, 1.74)	0.120	0.20	1.69e–18	1.00 (0.93, 1.09)	0.027	0.21	3.25e–19
1 400	1.01 (0.78, 1.44)	0.110	0.19	9.03e–17	1.00 (0.93, 1.09)	0.025	0.20	1.69e–18
1 600	1.01 (0.79, 1.45)	0.103	0.17	7.89e–13	1.00 (0.93, 1.10)	0.024	0.19	9.03e–17
1 800	1.00 (0.79, 1.37)	0.098	0.18	8.23e–15	1.00 (0.93, 1.10)	0.021	0.18	4.95e–14
2 000	1.00 (0.79, 1.43)	0.087	0.15	1.85e–10	1.00 (0.93, 1.07)	0.021	0.16	4.17e–12
2 500	1.00 (0.83, 1.37)	0.084	0.12	8.76e–07	1.00 (0.94, 1.07)	0.019	0.12	1.79e–06
2 700	1.00 (0.80, 1.33)	0.085	0.15	1.11e–09	1.00 (0.94, 1.06)	0.019	0.11	4.54e–06
2 800	1.01 (0.82, 1.38)	0.080	0.10	1.99e–04	1.00 (0.95, 1.05)	0.017	0.12	2.27e–06
3 000	1.00 (0.81, 1.32)	0.076	0.12	1.79e–06	1.00 (0.94, 1.06)	0.017	0.10	4.94e–05
4 000	1.00 (0.84, 1.27)	0.068	0.06	0.078	1.00 (0.96, 1.06)	0.014	0.06	0.038
5 000	1.00 (0.87, 1.26)	0.060	0.04	0.288	1.00 (0.95, 1.05)	0.013	0.03	0.759

*Note*: Results are shown for two social contact surveys (UK Social Contact Survey; POLYMOD) using two methods for estimating the reproduction number. The Kolmogorov–Smirnov statistic (K–S D.) and the associated *p*-value test whether the distribution at each sample size differs from the reference distribution at *n* = 5 800. Values are reported to two decimal places where appropriate.

At sample sizes below 200 individuals, the mixing matrix method produced R estimates ranging from 0.81 to 1.2 (2 s.f.), whereas the alternative individual-centred approach led to a wider range in estimates of the reproduction number (0.6–2.5; 2 s.f.).

The KS statistic – representing the maximum distance between cumulative distributions – decreases as sample size increases, illustrating that larger sample sizes result in greater accuracy and better representation of population uncertainty. The associated *p*-values support this; for low sample sizes, low *p*-values $<1\times 10^{-10}$ indicate that the R estimate distribution is not representative of the population.

With an individual-centred approach, for sample sizes of 1 000 participants, modelling estimates should be treated with caution as 63% of samples (626/1 000) resulted in a reproduction number within 10% of the true estimate. For 2 000 participants, 76% were within ±10% of the true estimate and for 3 000 participants, 84% of reproduction numbers were within ±10% .

Aggregating the data into larger age-bands reduces the sample size requirement. We show the impact of age band size in Supplementary Material section ‘Sensitivity to age band size’ and Supplementary Figure S4. Single-year age bands correspond most closely to the individual-centred approach used for the UKSCS, with population-level uncertainty decreasing as age band size increases. Estimated R values stabilize more rapidly with larger age aggregations. With 1 000 participants and 10-year age bands, 89% of estimates are within 5% of the true estimate.

For all methods considered, there were minimal differences in the resulting estimates when sample sizes greater than 3 000 individuals were considered.

## Discussion

We found considerable variability in the number of participants included in published social contact surveys, ranging from 30 to over 10 000 individuals, with a median sample size of 1 438. A quarter of surveys contained fewer than 1 000 participants. We show that small social contact surveys may result in inaccurate estimates of epidemic risk assessment and that very large surveys might be sampling unnecessarily. As a guide, sampling a minimum of 1 000 individuals is needed when conducting analysis for aggregate groups, whereas sampling over 2 000 individuals increases confidence in the derived results for a variety of model types. Sampling over 3 000 individuals did not improve model estimates.

Sample size estimates have been largely absent from the design of social contact surveys, despite their direct consequences for the reliability of transmission models. Existing systematic reviews have catalogued the diversity of survey methodologies and settings [1, 2], but none have addressed the statistical adequacy of the samples collected. Our findings provide a quantitative basis for evaluating published surveys and for measuring the uncertainty in epidemiological estimates. In particular, surveys containing fewer than 1 000 participants should be used with caution when parameterizing transmission models. Such smaller-scale surveys are often deployed to specific communities, for example, students [21] or farmers [22, 23]. Such populations may exhibit substantially different contact distributions than the general population and therefore have a different relationship between sample size and reproduction number uncertainty. In such cases, pilot studies and an exploration of this relationship are needed. Epidemic analyses and forecasts derived from contact survey data should be interpreted in the context of the sample size of the underlying surveys, and we recommend that sample sizes be reported as a standard element of all contact survey-derived epidemiological analyses.

The rapid review of social contact surveys provides a snapshot of current practice; however, it was restricted to English-language publications indexed in PubMed, and surveys reported in other languages or in grey literature were not captured. The rapid review approach meant that screening and data extraction were conducted by a single reviewer. We used the population-level mean reproduction number as our primary outcome metric; other quantities derived from contact data such as the critical vaccination threshold or age-specific incidence rates may show different sensitivity to sample size. Data quality represents a further constraint that is independent of sample size: survey fatigue, underreporting of high-contact days, and the systematic under-representation of certain demographic groups are well-documented in contact surveys and affect the validity of derived estimates in ways that cannot be remedied by increasing recruitment alone.

Our analysis was conducted using two social contact surveys from the UK and Europe which were designed to represent contacts in the general population. While the two surveys yielded broadly similar results, differences arose due to both survey design and methodology. The UKSCS explicitly asked about groups of individuals, which led to heavier tails, that is, with highly connected individuals occurring more often than expected [9, 10]. Sampling heavy-tailed networks can lead to underestimating the variance, more so when sampling without replacement; a study of heavy-tailed sexual networks suggested that sampling should be designed to capture this variability [24]. Heterogeneity in the underlying contact data is propagated into epidemic metrics using the individual-centred approach [16], retaining individual identity rather than aggregating individuals by age category [18].

In this analysis, we used the population-level mean reproduction number as our primary outcome metric, with finer model structure resulting in more variability and larger required sample sizes. The reproduction number is likely to exhibit greater uncertainty than other epidemic metrics such as the critical vaccination threshold or final epidemic size, as these quantities scale inversely with contact numbers. Further work could explore the propagation of uncertainty into alternative epidemic metrics and dynamic measures.

Our findings have implications for pandemic preparedness and response across Europe. Social contact data provide a leading indicator of the reproduction number, as they are more closely linked to transmission events than lagged indicators such as diagnoses, hospital admissions, or deaths. During the COVID-19 pandemic, social contact data were used to estimate the impact of physical distancing restrictions on transmission [6]. Our results highlight the vulnerability of such early estimates to study design and sample size and suggest that further work is needed to tailor sampling schemes to capture highly connected individuals. Establishing a minimum sample size for social contact surveys will improve comparability between settings, supporting more consistent epidemic intelligence. This study provides the first exploration of sample size and R uncertainty, enabling more reliable estimation of R and strengthening the evidence base for coordinated public health decision-making.

## Supporting information

10.1017/S095026882610212X.sm001 Brooks-Pollock and Danon supplementary material Brooks-Pollock and Danon supplementary material

## Data Availability

Code was written using R Studio, R version 4.5.3 (2026-03-11). All data used in this article are anonymous and available in the public domain. POLYMOD and the Hong Kong social contact data are available from https://socialcontactdata.org/ and the UKSCS data from http://wrap.warwick.ac.uk/54273/. Processed data and code are available at github.com/bristol-vaccine-centre/contact-sample-size.

## Supplementary material

The supplementary material for this article can be found at http://doi.org/10.1017/S095026882610212X.

## Long descriptions

### Figure 1. Long description

A histogram of social contact survey sample sizes from a rapid review of 107 surveys. The horizontal axis runs from 0 to 10 000 and the vertical axis is marked with 0, 5, and 10 counts. The data are binned into units of 200. Four surveys contained fewer than 200 participants and there are five surveys that exceeded 10 000. The distribution is right skewed, with most surveys containing fewer than 2 500 participants. The median sample size is 1 438 participants.

Navigate back to Figure 1..

### Figure 2. Long description

The visualization consists of four panels labeled A through D.

Panel A (top-left) is a scatter plot. The x-axis represents Sample size from 0 to 6000. The y-axis represents Normalised R from 0.8 to 1.4. The data shows a funnel-shaped distribution where the variance of Normalised R is high at small sample sizes and converges toward a value of 1.0 as the sample size increases.

Panel B (top-right) is a density plot corresponding to the data in Panel A. The x-axis is Density from 0 to 30, and the y-axis is Normalised R from 0.8 to 1.4. Multiple colored curves represent different sample sizes. As sample size increases, the curves become narrower and taller, peaking sharply at Normalised R equals 1.0.

Panel C (bottom-left) is a scatter plot similar to Panel A but for a different dataset. The x-axis is Sample size from 0 to 6000, and the y-axis is Normalised R from 0 to 4. The data shows a similar convergence toward 1.0, but with much higher initial variance and outliers reaching up to 4 at low sample sizes.

Panel D (bottom-right) is a density plot for the data in Panel C. The x-axis is Density from 0 to 6, and the y-axis is Normalised R from 0 to 4. The curves show a similar narrowing trend toward 1.0 as sample size increases, though the density values are lower than in Panel B.

A legend on the far right titled Sample size lists nine color-coded categories: 100 (red), 500 (orange), 1000 (olive), 1500 (green), 2000 (teal), 3000 (light blue), 4000 (blue), and 5000 (pink).

Navigate back to Figure 2..

### Table 1. Long description

The table provides statistics (mean, min, max, standard deviation, KS distance, and p-value) for normalised R values according to sample size (ranging from 200 to 5,000). The results are shown for the UK Social Contact Survey and POLYMOD. The mean is always close to 1, but the minimum increases with sample size, and the maximum decreases. The standard deviation, KS statistic and p-value decrease with sample size.

Navigate back to Table 1..
