# Supplementary material for: Sample size in social contact surveys for epidemic modelling and risk prediction
Source: Epidemiol Infect. 2026 Oct 15;154:e131. doi: 10.1017/S095026882610212X (PMC13749101; doi:10.1017/S095026882610212X)
Supplement: Supplementary file 1 [file S095026882610212Xsup001.docx]

**SUPPLEMENTARY INFORMATION**

**Sample size in social contact surveys for epidemic modelling and risk prediction**

Ellen Brooks-Pollock & Leon Danon

Table of Contents

[1.	Rapid review PRISMA flow diagram	2](#_Toc234226279)

[2.	Application to other settings	3](#_Toc234226280)

[a.	Implementation for Hong Kong social contact survey	3](#_Toc234226281)

[b.	Implementation to other settings	4](#_Toc234226282)

[3.	Sensitivity analysis	5](#_Toc234226283)

[a.	Sampling with and without replacement	5](#_Toc234226284)

[b.	Sensitivity to age band size	6](#_Toc234226285)

[4.	References	7](#_Toc234226286)

# 1. Rapid review PRISMA flow diagram

The rapid review described in the main manuscript was conducted and reported in accordance with the PRISMA 2020 Statement guidelines(1). The PRISMA flow diagram summarising the identification, screening, eligibility assessment and inclusion of studies is provided in figure S1 below .

Figure S1: PRISMA 2020 flow diagram describing the identification, screening, eligibility assessment and inclusion of studies in the rapid review.

# 2. Application to other settings

## a. Implementation for Hong Kong social contact survey

To assess the generalisability of our findings, we repeated the analysis using the Hong Kong social contact survey conducted in 2015–2016 by Leung et al.(2). This survey, which was based on the POLYMOD study design, included 557 male participants and 592 female participants.

Figure S2 shows the relationship between survey sample size and the normalised dominant eigenvalue of the next-generation matrix, which provides a measure proportional to the basic reproduction number (*R*). Unlike the UK Social Contact Survey analysed in the main manuscript, the distribution of estimated values does not fully stabilise within the available sample size. This finding is consistent with the conclusions of the main analysis, which suggest that over 1,000 participants are required to obtain stable and robust estimates of *R* from social contact surveys.

Figure S2: Relationship between the number of survey participants and the estimated reproduction number, measured as the normalised dominant eigenvalue of the next-generation matrix, using data from the 2015–2016 Hong Kong social contact survey.

## b. Implementation to other settings

The GitHub repository <https://github.com/bristol-vaccine-centre/contact-sample-size> contains the R code used to investigate the relationship between social contact survey sample size and the stability of epidemic metrics, including the dominant eigenvalue of the next-generation matrix and the effective reproduction number. The analyses reproduce the figures, tables and supplementary analyses presented in the manuscript.

POLYMOD-style data: The code requires social contact data of the format used by the <https://socialcontactdata.org/> data repository. The required datasets and variables are: participant_common: part_id and part_age and contact_common: part_id and cnt_age. The analysis will construct age-specific contact matrices using 10-year age bands and calculates the dominant eigenvalue of the resulting matrix.

Other format contact data: The minimum data required is a degree distribution, from which a per-person individual reproduction number can be calculated.

# 3. Sensitivity analysis

## a. Sampling with and without replacement

The analyses presented in the main manuscript used sampling with replacement when repeatedly drawing subsets of participants from the original survey data. This approach reflects the interpretation that the observed survey participants represent a sample from a much larger underlying population.

An alternative approach is to sample without replacement, which ensures that each participant is selected at most once in each resampled dataset. Sampling without replacement effectively increases the information contained within each sample and therefore results in a larger effective sample size than sampling with replacement.

To evaluate the effect of this modelling choice, we repeated the analyses using both the UK Social Contact Survey and the POLYMOD dataset with sampling performed without replacement. The corresponding results are presented in figure S3.

The overall conclusions are unchanged. In particular, the estimated sample size required to obtain stable estimates of the reproduction number remains similar to that reported in the main manuscript. As expected, sampling with replacement produces slightly greater variability in the estimated reproduction numbers because individual participants may be selected multiple times within the same resampled dataset.

Figure S3: Sample size results using without replacement sampling for comparison with sampling with replacement as used in the main paper.

## b. Sensitivity to age band size

The construction of age-stratified contact matrices requires the definition of age groups, and different studies commonly use different age-band widths. To assess the sensitivity of our results to this choice, we repeated the analyses using the POLYMOD dataset with alternative age stratifications.

We compared contact matrices constructed using 1, 5, 10 and 20-year age bands. The resulting estimates of the normalised reproduction number were qualitatively similar across age stratifications. Single year age bands produced the greatest variability in R estimates (top left panel, Fig. S4), with variability decreasing as greater numbers of individuals were aggregated into larger age bands. The single year age group model was most similar to the individual-centred approach using the UK Social Contact Survey.

Figure S4: Sensitivity of estimated reproduction numbers to the age-band width used to construct the contact matrix. Results compare contact matrices based on 1-(top left), 5-(top right), 10-(bottom left) and 20-(bottom right) year age groups using the POLYMOD dataset.

# 4. References

1.	Page MJ, McKenzie JE, Bossuyt PM, Boutron I, Hoffmann TC, Mulrow CD, et al. The PRISMA 2020 statement: an updated guideline for reporting systematic reviews. BMJ. 2021 Mar 29;372:n71. doi:10.1136/bmj.n71 PubMed PMID: 33782057.

2.	Leung K, Jit M, Lau EHY, Wu JT. Social contact patterns relevant to the spread of respiratory infectious diseases in Hong Kong. Sci Rep. 2017 Aug 11;7(1):7974. doi:10.1038/s41598-017-08241-1
